# *Helicobacter fennelliae* Localization to Diffuse Areas of Human Intestine, Japan

**DOI:** 10.3201/eid3001.231049

**Published:** 2024-01

**Authors:** Takashi Sakoh, Emiko Miyajima, Yusuke Endo, Kei Kono, Junichiro Sato, Mizuki Haraguchi, Sho Ogura, Masayo Morishima, Keiko Ishida, Yorinari Ochiai, Shu Hoteya, Yutaka Takazawa, Masaru Baba, Hideki Araoka

**Affiliations:** Toranomon Hospital, Tokyo, Japan

**Keywords:** *Helicobacter fennelliae*, human, intestine, infection, colon localization, bacteremia, bacteria, Japan

## Abstract

The site of enterohepatic *Helicobacter* colonization/infection in humans is still unknown. We report microbiologically and histopathologically confirmed *H. fennelliae* localization in the large intestine in an immunocompromised patient in Japan. This case contributes to better understanding of the life cycle of enterohepatic *Helicobacter* species.

*Helicobacter* species are classified into enterohepatic and gastric species. Human infections with enterohepatic species have been reported mainly as bloodstream infections caused by *H. cinaedi* and *H. fennelliae* in the immunocompromised patients (*1*–*3*). Intestinal localization and bacterial translocation have been suggested as the origin of those infections (*4*,*5*) because the same pathogen is detected in the stool of patients with *Helicobacter* bacteremia (*3*,*6*). However, evidence is limited regarding where enterohepatic *Helicobacter* is present in the gastrointestinal tract. We describe a confirmed human case of localization of *H. fennelliae* in crypts of the mucosal epithelium of the large intestine in a patient with recurrent *H. fennelliae* bacteremia.

## The Case Study

**Figure 1:**
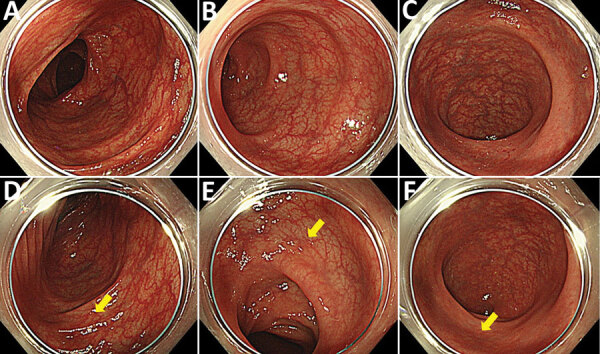
Colonoscopy findings from a man in Japan who had recurrent *Helicobacter fennelliae* bacteremia. A–C) First colonoscopy findings in the transverse colon (A), in the sigmoid colon (B), and in the rectum (C). D–F) Second colonoscopy findingsin the transverse colon (D), in the sigmoid colon (E), and in the rectum (F). Yellow arrows indicate randomly biopsied sites. Colonic vascular permeability was preserved, and there were no significant findings for inflammation.

A 62-year-old man sought treatment at Toranomon Hospital, Tokyo, Japan, for recurrence of *H. fennelliae* bacteremia, causing cellulitis of the left lower leg. He had received 3 weeks of intravenous ampicillin therapy, followed by oral doxycycline therapy, for the first episode *H. fennelliae* bacteremia, which initially resulted in a confirmed negative blood culture. However, blood culture became positive again under oral doxycycline therapy. *H. fennelliae* could be detected in stool culture before ampicillin administration, which raised suspicion of entry via the intestinal tract. However, the most recent colonoscopy (first colonoscopy) was performed before the first episode of bacteremia 5.5 weeks earlier, which showed no abnormal findings other than two 3-mm erosions in the cecum (biopsies revealed no abnormal tissue) and some small-sized adenomatous lesions in the colon (Figure 1, panels A–C). A fourth-generation HIV antigen/antibody combination assay was negative. 

The blood tests we conducted at the time of recurrence showed hypogammaglobulinemia (IgG 625 mg/dL, IgA 38.6 mg/dL, IgM 1.0 mg/dL), leading us to suspect impaired intestinal immunity, which prompted a repeat colonoscopy. Gastroenterologists performed a second colonoscopy to observe the mucosal surface, taking random biopsies and tissue cultures of the cecum, ascending colon, transverse colon, descending colon, sigmoid colon, and rectum. Pathologists assessed the biopsy tissue by using hematoxylin-eosin and Warthin-Starry silver staining. Microbiologists homogenized cultured tissue specimens and suspended them in saline, and infectious disease specialists assessed Gram staining of the suspension. Laboratory staff incubated microaerobic cultures of the tissue suspension in modified Skirrow medium EX (Shimadzu Diagnostics Corporation, https://corp.sdc.shimadzu.co.jp) at 35°C. After the bacterial colony was obtained, we confirmed the pathogen by using matrix-assisted laser desorption/ionization time-of-flight (MALDI-TOF) mass spectrometry (Microflex LT, flex control 3.4.135.0, MALDI Biotyper 4.1.1; Bruker Daltonics, https://www.bruker.com/en.html) (Appendix). 

**Figure 2:**
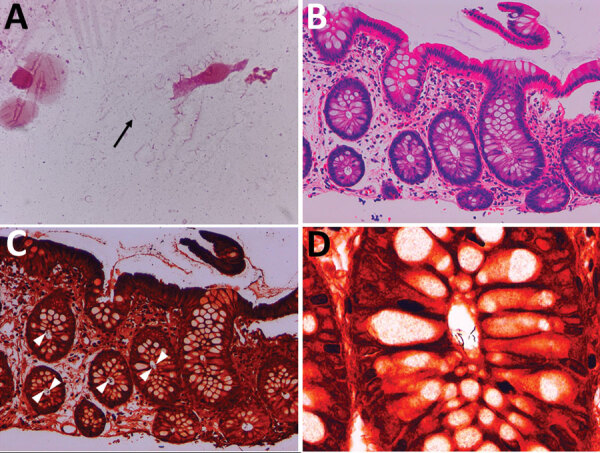
Microscopic findings in ileocolonic biopsy specimens (second colonoscopy) in a man in Japan who had recurrent *Helicobacter fennelliae* bacteremia. A) Morphologic features of the bacteria in a cecal tissue suspension with Gram staining (original magnification ×2,000). Arrow indicates gram-negative spiral bacilli. B) Histologic findings in a biopsy specimen taken from the transverse colon with hematoxylin-eosin staining (original magnification ×200). The colonic mucosa shows mild leukocytic infiltration. C) Histologic findings in a biopsy specimen taken from the transverse colon with Warthin-Starry silver staining (original magnification ×200). Bacteria are aggregated in crypts (arrowheads) D) Morphologic features of bacteria obtained from the transverse colon with Warthin-Starry silver staining (original magnification ×1,000). A cluster of spiral bacilli was observed.

Colonoscopy confirmed that the erosions in the cecum seen at the time of the previous examination had disappeared. Overall, the colonic vascular permeability was preserved and there were no erosions, ulcers, or other findings suspicious for inflammation (Figure 1, panels D–F). Gram staining showed gram-negative spiral bacilli in the tissue obtained from the cecum and transverse colon (Figure 2, panel A). Hematoxylin and eosin staining of the biopsied tissues showed only slight inflammation with very mild edema of the mucosa in both cases, preservation of goblet cells, and mild leukocytic infiltration. In contrast, Warthin-Starry silver staining showed bacteria with spiral structures in crypts of the cecum, ascending colon, transverse colon, descending colon, and sigmoid colon but not in the rectal tissues (Figure 2, panels B–D). Culture of the tissue suspension showed bacterial growth in all tissues from the cecum, the ascending, transverse, descending, and sigmoid colon, and the rectum, which we identified as *H. fennelliae* by MALDI-TOF mass spectrometry, confirming the localization of *H. fennelliae*.

## Conclusions

This study provides evidence for the localization of *H. fennelliae* in the intestinal tract. This confirmation may lead to better understanding of the life cycle of enterohepatic *Helicobacter* species. Reports of human infections with *H. cinaedi* are scarce but are gradually increasing (*3*–*5*), whereas those of *H. fennelliae* are even scarcer (*2*). Enterohepatic *Helicobacter* species cannot be identified based on biochemical characteristics alone. A definitive diagnosis requires identification by PCR, MALDI-TOF mass spectrometry, or both (*3*,*7*). Detection from stool and tissue is also difficult by routine culture methods, and many cases might have been overlooked (*3*).

Some previous reports have focused on disturbance of the intestinal tract by enterohepatic *Helicobacter* in animal models, but not in humans (*8*). Experiments conducted on pigtailed macaques given *H. cinaedi* and *H. fennelliae* orally showed that diarrhea, bacteremia, and localized inflammation of the colon were seen 3–7 days after exposure and that organisms remained detectable in stools for 3 weeks after diarrhea resolved. Researchers noted no acute inflammatory findings or microbial adherence in the small or large intestine in animals in the *H. cinaedi* group; however, they did note lymphoid hyperplasia caused by immune stimulation in the Peyer’s patches. In contrast, our case of a human patient with recurrent bacteremia showed broad localization of *H. fennelliae* in the crypts of the colonic mucosa, almost without background inflammation in the intestinal tract or grossly visible disruption of the mucosal barrier.

The lack of inflammation and mucosal abnormality may reflect the fact that the patient was immunocompromised with hypogammaglobulinemia and lacked adequate intestinal immunity, which possibly caused bacteremia. Fujiya et al. described a patient with *H. fennelliae* bacteremia who had been receiving anticancer chemotherapy that included carboplatin, a possible risk factor of mucositis (*9*). They mentioned the possibility of the damaged intestinal mucosa being the route of entry for the pathogen. However, retrospective Warthin-Starry silver staining of the biopsied erosive mucosal tissue in the cecum from the first colonoscopy in the patient we describe showed no spiral bacillus-like structures in the mucosal lesions. Furthermore, we detected no gross abnormalities in the second colonoscopy. Given the absence of abnormal mucosal findings in our patient, immunoglobulin-associated impairment of intestinal immunity may be more important than damage to the intestinal mucosa in prevention of pathogen entry. This hypothesis would explain the mechanism caused in a previously reported patient with X-linked agammaglobulinemia, who developed recurrent bacteremia with persistent detection of *H. cinaedi* in stools despite repeated courses of antimicrobial therapy and selective decontamination of the digestive tract (*10*).

We report the microbiological and histopathological confirmation of *H. fennelliae* localization in the large intestine in an immunocompromised patient. Only 1 other report, regarding a patient in Japan, had enterohepatic *Helicobacter* detected pathologically (*11*). That patient contracted *Clostridioides difficile* enteritis after treatment for *H. cinaedi* bacteremia that resulted in bloody stools. Colonoscopy revealed pseudomembranous mucosal tissue in the cecum, where *H. cinaedi* was detected and confirmed pathologically. The authors stated, however, that it was impossible to determine whether the enteritis found in their patient was caused by *C. difficile* or *H. cinaedi* and whether it was localized at the time of the onset of *H. cinaedi* bacteremia. In our case, *H. fennelliae* was already present in stool culture at the time of the initial episode of bacteremia. Moreover, when the recurrent bacteremia was detected, persistence of the localized pathogen in the intestinal tract after the resolution of the first episode of bacteremia was confirmed. Both results suggest entry of *H. fennelliae* via the intestinal tract. We believe that investigating carriage rate or pathogenicity of enterohepatic *Helicobacter* in humans will help to better establish the disease concept.

Appendix More information is available for Helicobacter fennelliae localization to diffuse areas of human intestine.
