# Supplementary material for: Helicobacter fennelliae Localization to Diffuse Areas of Human Intestine, Japan
Source: Emerg Infect Dis. 2024 Jan;30(1):129–32. doi: 10.3201/eid3001.231049 (PMC10756365; doi:10.3201/eid3001.231049)
Supplement: Appendix — More information is available for Helicobacter fennelliae localization to diffuse areas of human intestine. [file 23-1049-Techapp-s1.pdf]

Article DOI: http://doi.org/10.3201/eid3001.231049

EID cannot ensure accessibility for supplementary materials supplied by authors. Readers who have difficulty accessing supplementary content should contact the authors for assistance.

Helicobacter fennelliae Localization to Diffuse Areas of Human Intestine

Appendix

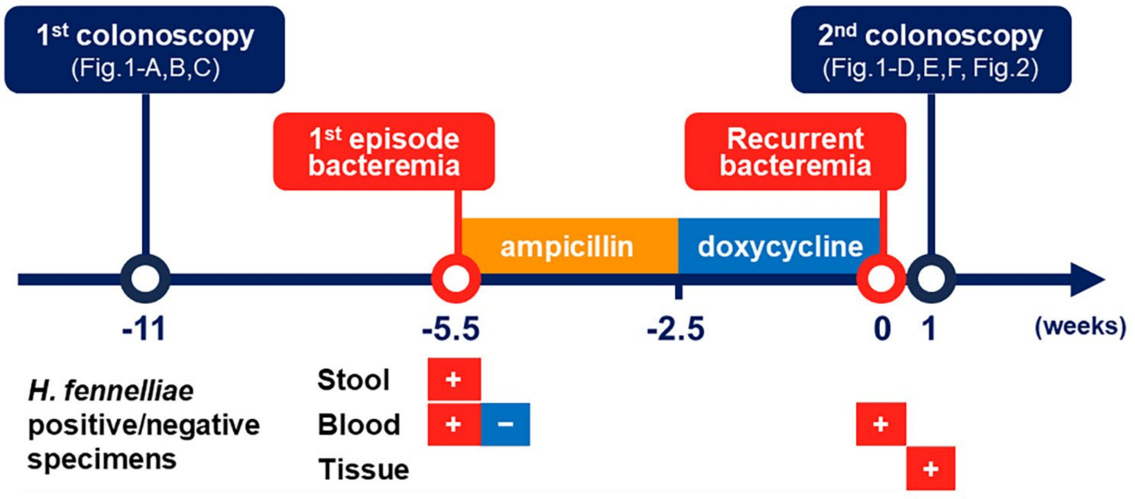

Appendix Figure. A timeline of the events associated with a patient with localization of *Helicobacter fennelliae* to the large intestine.
